# Supplementary material for: Non-Toxigenic Clostridioides difficile Strain E4 (NTCD-E4) Prevents Establishment of Primary C. difficile Infection by Epidemic PCR Ribotype 027 in an In Vitro Human Gut Model
Source: Antibiotics (Basel). 2023 Feb 22;12(3):435. doi: 10.3390/antibiotics12030435 (PMC10044524; doi:10.3390/antibiotics12030435)
Supplement: Supplementary file 1 [file antibiotics-12-00435-s001.zip › antibiotics-2166842-supplementary.pdf]

A

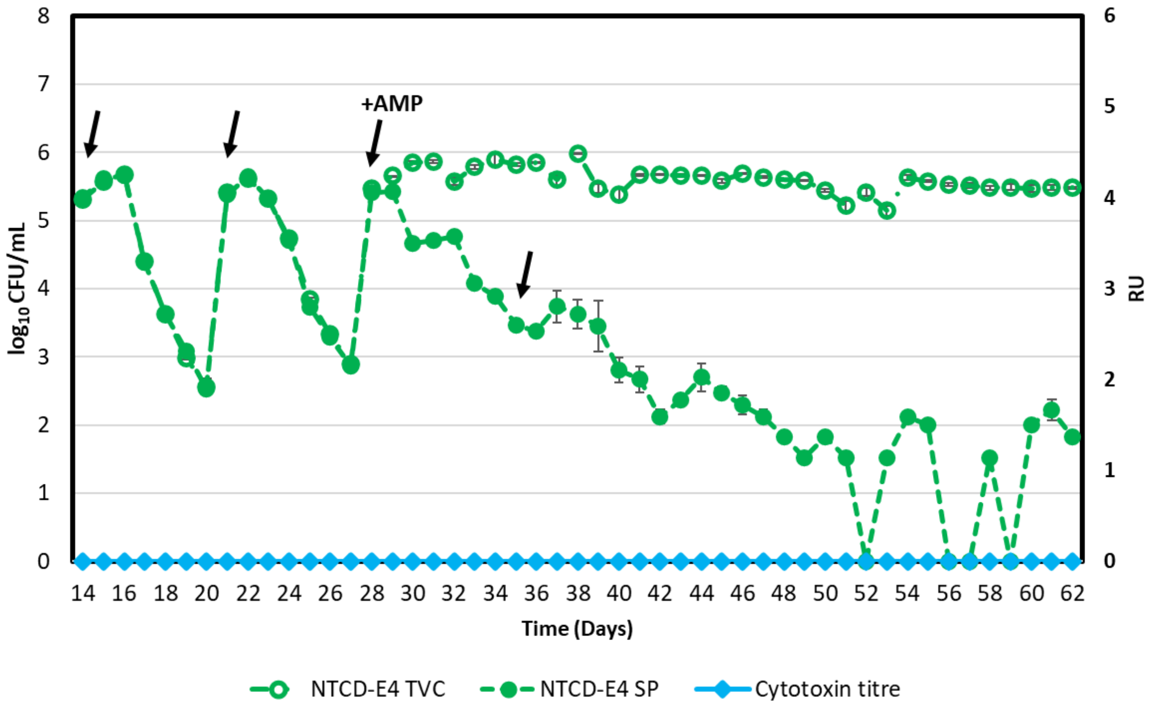

B

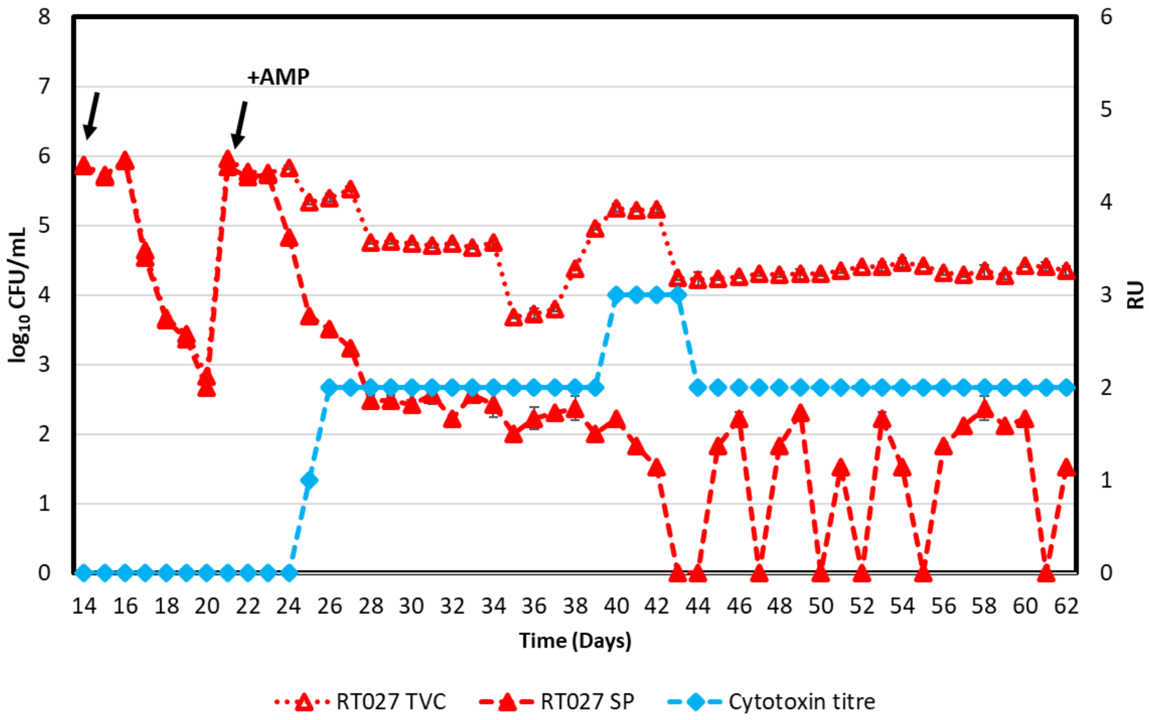

Figure S1. Effect of ampicillin (AMP, 8mg/L, 8-hourly) on *C. difficile* strains, NTCD-E4 and RT027, total viable counts (TVC), spore counts (SP), and cytotoxin titres (RU) in a human gut model (Vessel 3, pH 6.8). Brazier's agar was used to culture all *C. difficile* strains, incorporating tetracycline (TET, 0.5mg/L) for RT027 and clindamycin (DA, 8 mg/L) for NTCD-E4. Spore counts were determined following and alcohol shock and cytotoxin titres (Relative Units, RU) were determined using a Vero cell cytotoxicity assay. A, monoculture experiment containing NTCD-E4; B, monoculture experiment containing RT027. Viable count data are log_{10}CFU/mL ±SE. Arrows indicate *C. difficile* spore inoculation points.

A

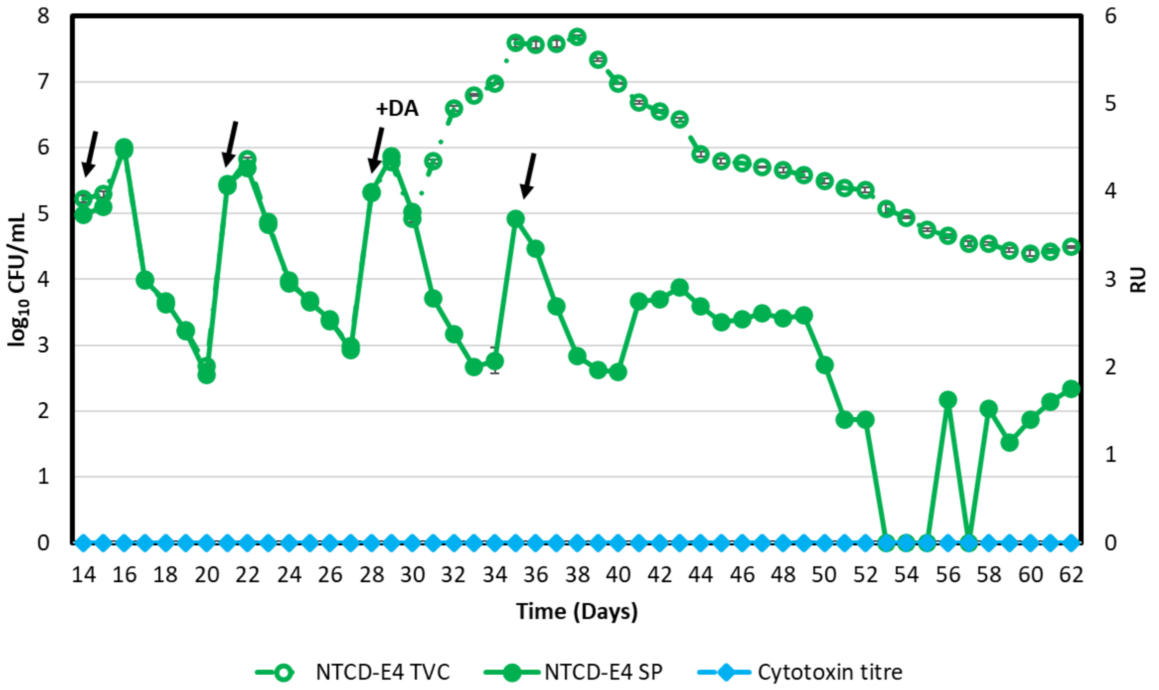

B

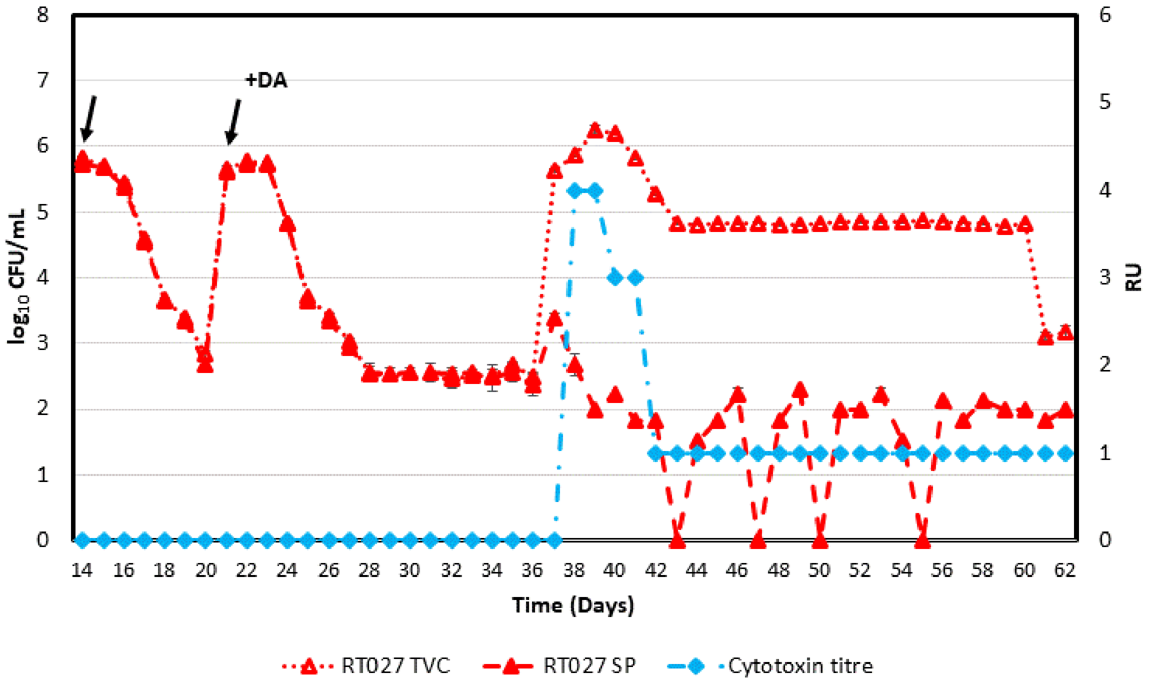

Figure S2. Effect of clindamycin (DA, 33.9 mg/L, 6-hourly) on *C. difficile* strains, NTCD-E4 and RT027, total viable counts (TVC), spore counts (SP), and cytotoxin titres in a human gut model (Vessel 3, pH 6.8). Brazier's agar was used to culture all *C. difficile* strains, incorporating tetracycline (TET, 0.5mg/L) for RT027 and clindamycin (DA, 8 mg/L) for NTCD-E4. Spore counts were determined following and alcohol shock and cytotoxin titres (Relative Units, RU) were determined using a Vero cell cytotoxicity assay. A, monoculture experiment containing NTCD-E4; B, monoculture experiment containing RT027. Viable count data are log_{10}CFU/mL ±SE. Arrows indicate *C. difficile* spore inoculation points.

A

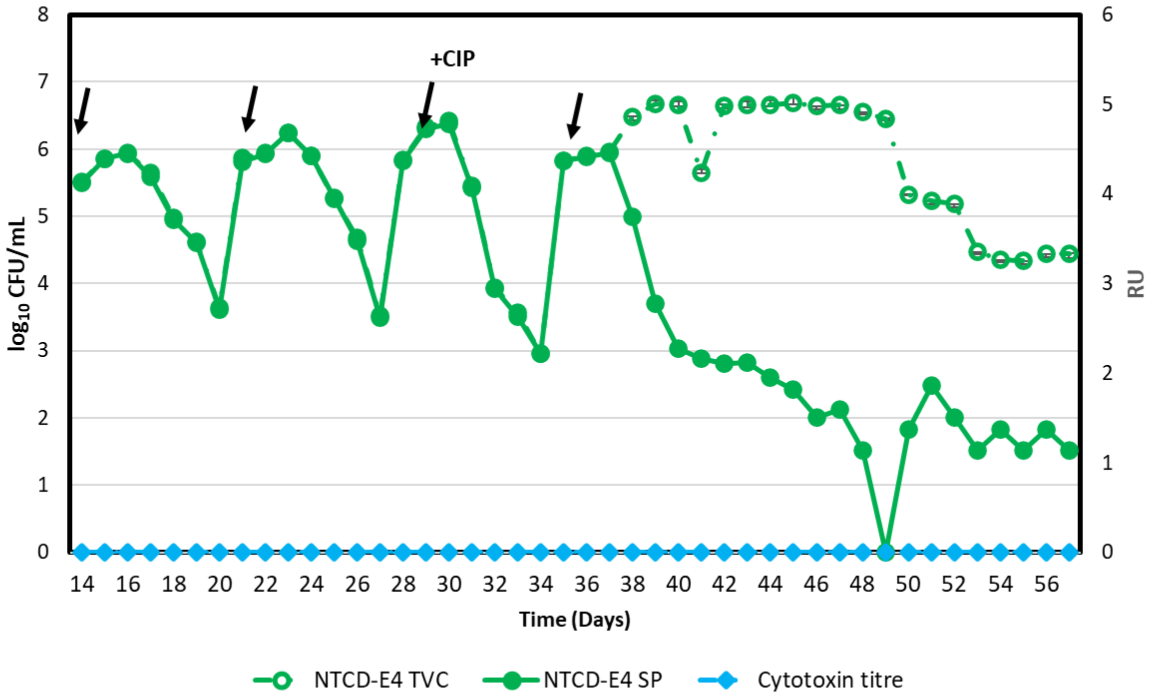

B

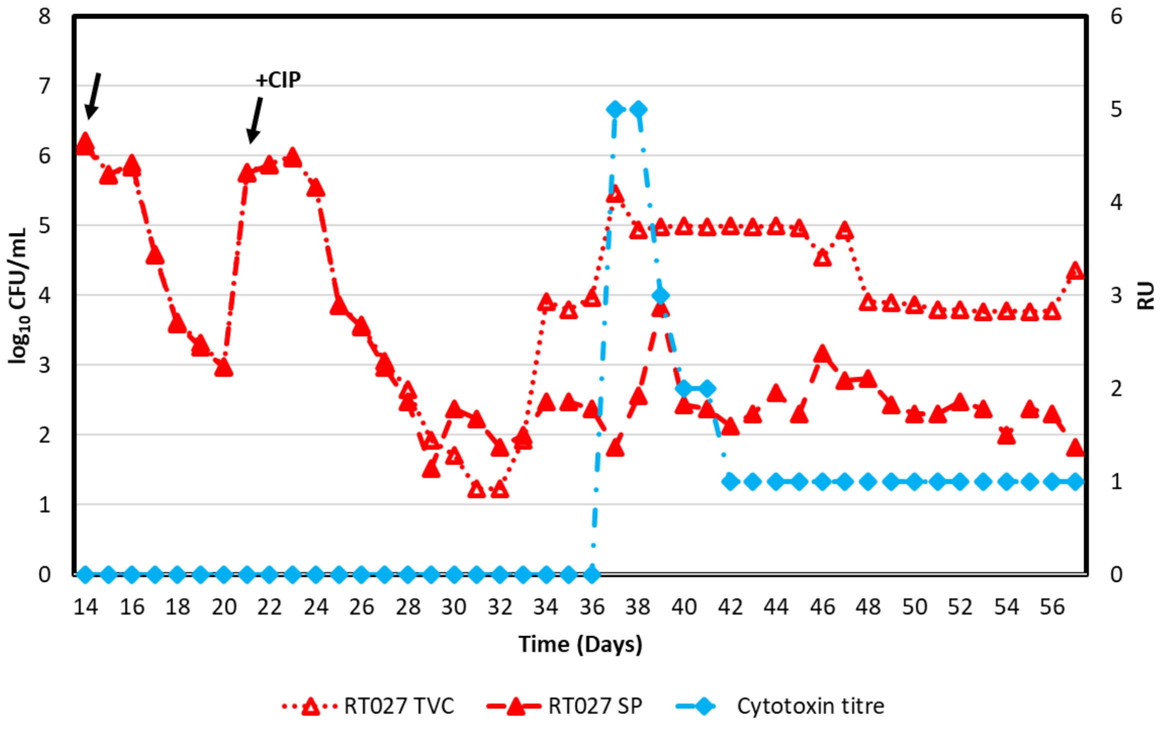

Figure S3. Effect of ciprofloxacin (CIP, 139 mg/L, 12-hourly) on *C. difficile* strains, NTCD-E4 and RT027, total viable counts (TVC), spore counts (SP), and cytotoxin titres in a human gut model (Vessel 3, pH 6.8). Brazier's agar was used to culture all *C. difficile* strains, incorporating tetracycline (TET, 0.5mg/L) for RT027 and clindamycin (DA, 8 mg/L) for NTCD-E4. Spore counts were determined following and alcohol shock and cytotoxin titres (Relative Units, RU) were determined using a Vero cell cytotoxicity assay. A, monoculture experiment containing NTCD-E4; B, monoculture experiment containing RT027. Viable count data are log_{10}CFU/mL ±SE. Arrows indicate *C. difficile* spore inoculation points.

A

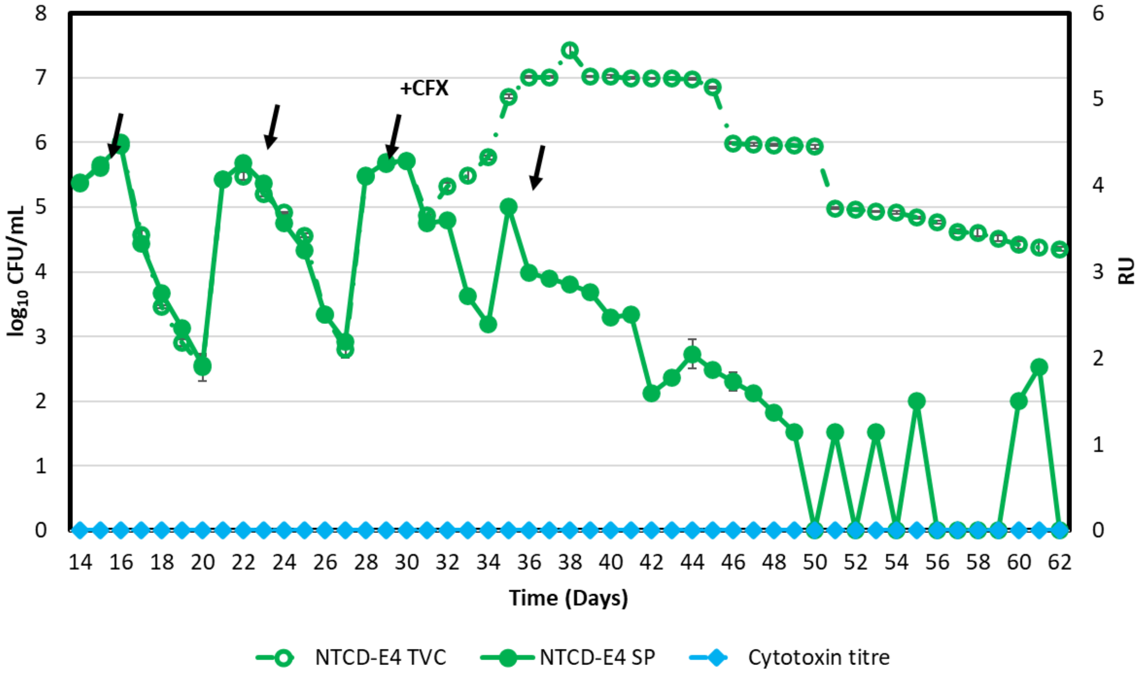

B

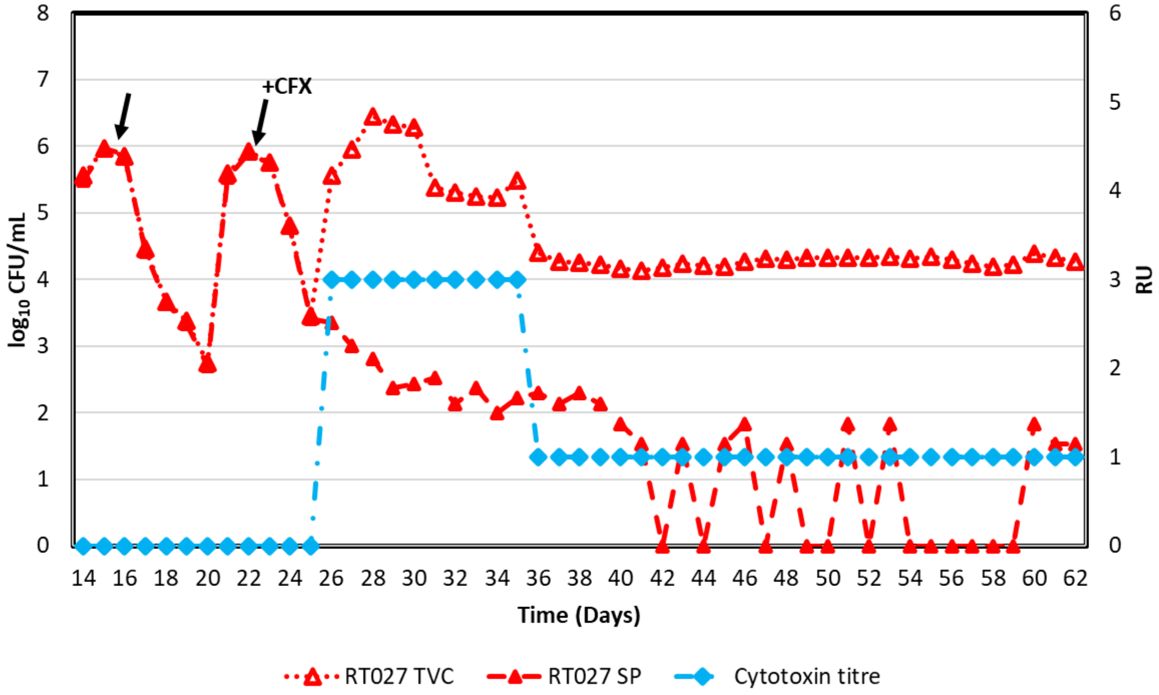

Figure S4. Effect of cefotaxime (CFX, 20 mg/L, 12-hourly) on *C. difficile* strains, NTCD-E4 and RT027, total viable counts (TVC), spore counts (SP), and cytotoxin titres (CYT) in a human gut model (Vessel 3, pH 6.8). Brazier's agar was used to culture all *C. difficile* strains, incorporating tetracycline (TET, 0.5mg/L) for RT027 and clindamycin (DA, 8 mg/L) for NTCD-E4. Spore counts were determined following and alcohol shock and cytotoxin titres (Relative Units, RU) were determined using a Vero cell cytotoxicity assay. A, monoculture experiment containing NTCD-E4; B, monoculture experiment containing RT027. Viable count data are log_{10}CFU/mL ±SE. Arrows indicate *C. difficile* spore inoculation points.
